# Mental Health Challenges of United States Healthcare Professionals During COVID-19

**DOI:** 10.3389/fpsyg.2020.02065

**Published:** 2020-08-13

**Authors:** Ann Pearman, MacKenzie L. Hughes, Emily L. Smith, Shevaun D. Neupert

**Affiliations:** ^1^School of Psychology, Georgia Institute of Technology, Atlanta, GA, United States; ^2^Department of Psychology, North Carolina State University, Raleigh, NC, United States

**Keywords:** health care professionals, pandemic (COVID-19), stress and coping, depression, anxiety

## Abstract

As COVID-19 continues to impact global society, healthcare professionals (HCPs) are at risk for a number of negative well-being outcomes due to their role as care providers. The objective of this study was to better understand the current psychological impact of COVID-19 on HCPs in the United States This study used an online survey tool to collect demographic data and measures of well-being of adults age 18 and older living in the United States between March 20, 2020 and May 14, 2020. Measures included anxiety and stress related to COVID-19, depressive symptoms, current general anxiety, health questions, tiredness, control beliefs, proactive coping, and past and future appraisals of COVID-related stress. The sample included 90 HCPs and 90 age-matched controls (*M*_age_ = 34.72 years, *SD* = 9.84, range = 23 – 67) from 35 states of the United States. A multivariate analysis of variance was performed, using education as a covariate, to identify group differences in the mental and physical health measures. HCPs reported higher levels of depressive symptoms, past and future appraisal of COVID-related stress, concern about their health, tiredness, current general anxiety, and constraint, in addition to lower levels of proactive coping compared to those who were not HCPs (*p* < 0.001, η^2^ = 0.28). Within the context of this pandemic, HCPs were at increased risk for a number of negative well-being outcomes. Potential targets, such as adaptive coping training, for intervention are discussed.

## Introduction

On May 14, 2020, the United States had 1,340,098 confirmed COVID-19 cases with 80,695 deaths (World Health Organization, 2020) and was considered the epicenter of the pandemic. Although social distancing and quarantine guidelines have slowed the pandemic’s spread, the recent relaxing of guidelines suggests continued challenges to the healthcare systems and healthcare professionals (HCPs). Indeed, there are calls for COVID-19 to be considered as a new occupational hazard for H around the globe (Godderis et al., 2020). Not only are many HCPs more likely to be exposed to and, therefore, contract COVID-19, but providing care during a pandemic can place tremendous pressure on HCPs caring for very sick and dying patients, helping the families of the sick, and dealing with the frustrations of healthcare systems, all while trying to take care of their own families and loved ones (Maunder et al., 2003; Bai et al., 2004).

Studies out of China have examined the experiences of HCPs during the height of their COVID-19 outbreak. In a sample of 1,563 medical staff workers in China working during the COVID-19 pandemic, 73.4% reported stress-related symptoms, 50.7% reported symptoms of depression, 44.7% reported anxiety, and 36.1% reported experiencing insomnia (Liu et al., 2020). Lai et al. (2020) found evidence for higher rates of anxiety, depression, and distress among HCPs in Wuhan compared to HCPs in other regions in China. Other studies examined the need for and impact of services offered to healthcare workers, such as adjusting shifts to allow time for rest (Chen et al., 2020; Kang et al., 2020).

While there have been several well-written opinion pieces and commentaries regarding the well-being of healthcare workers in the United States during this pandemic (Godderis et al., 2020; Gold, 2020; Greenberg et al., 2020), we are aware of only one descriptive study with data from New York City (Shechter et al., 2020) that did not include a control group. There have been several meta-analyses and reviews of the impact of this pandemic on HCPs internationally (Chew et al., 2020; Pappa et al., 2020; Rajkumar, 2020), but no studies from the United States were available to be included in these studies. Previous studies have shown that the mental health challenges HCPs face during pandemics often impact their ability to continue to be part of the frontlines working to help treat and care for patients and their own families (Maunder et al., 2006; Shechter et al., 2020). Further, enduring psychological effects could negatively impact their ability to provide patient care in the future as well as impacting their quality of life (Goulia et al., 2010). A crucial mission for researchers during this time is enhancing our understanding of the experiences of HCPs in order to plan for interventions and care both in the short-term (now) and in the long-term (over the next couple of years). The current study is designed to examine several critical outcomes such as depressive symptoms, anxiety (current general anxiety as well as anxiety about developing COVID-19), COVID-related stress, and health in HCPs during the early months of the COVID-19 pandemic across the entire United States. In addition, we also examine potentially beneficial indicators of resilience such as control beliefs and proactive coping.

Psychiatric morbidity in the forms of depression and/or anxiety not only is troubling in its own right, but is also highly correlated with burnout, higher rates of chronic diseases, reduced quality of life, and suicide (Kumar, 2016). During the severe acute respiratory syndrome (SARS) pandemic in Goulia et al. (2010) found that the pressure of the work environment combined with fears about the disease itself created negative outcomes in the form of anxiety and depression that had profound impacts on the well-being of healthcare workers during that time. Additionally, follow-up studies revealed that the emotional distress from the pandemic was often long-lasting (Maunder et al., 2006). For instance, one to 2 years after the SARS outbreak, Maunder et al. (2006) found that SARS healthcare workers reported higher levels of burnout and distress, had increased smoking and alcohol consumption, were more likely to have reduced patient contact, and worked fewer hours compared to healthcare workers who did not treat SARS. The SARS outbreak was much more contained than the current worldwide pandemic which has even greater potential to have both ongoing and lasting consequences on society as a whole and HCPs in particular.

Identifying opportunities for resilience will be especially critical to combat the negative consequences. Control beliefs represent the subjective perceptions that one can influence what happens in one’s life and include beliefs or expectations about the extent to which one’s actions can bring about desired outcomes (Agrigoroaei and Lachman, 2010). Lachman and Firth (2004) distinguished two main sources of control: one’s own efficacy (internal control, competence, or personal mastery), and the responsiveness of the environment or other people (external control, contingency, or perceived constraints) (Bandura, 1977). The two control beliefs included in the present study are mastery and constraint. Mastery is often described in terms of one’s judgments about his or her ability to achieve a goal, while perceived constraints refers to the extent to which people believe factors exist which interfere with goal attainment (Lachman and Weaver, 1998b). Pearlin and Schooler (1978) suggested that personal mastery is an important psychological resource that mitigates the effects of stress and strain, and it is also associated with reduced reactivity to work-related stressors (Neupert et al., 2007). When faced with stressful situations, a strong sense of control has also been linked to low levels of self-reported perceived stress (Cameron et al., 1991) and lower risk of depression (Yates et al., 1999).

Aspinwall and Taylor (1997) characterized proactive coping as a series of steps one takes to preemptively modify or avoid stressful events. Those who have higher levels of proactive coping compared to those with lower levels of proactive coping have more meaning in life (Miao et al., 2017), fewer symptoms of PTSD (Vernon et al., 2009), and higher levels of quality of life (Cruz et al., 2018). Proactive coping is also associated with lower levels of depression, fewer declines in functional disability in aging, and larger systems of social support (Greenglass et al., 2006; Bokszczanin, 2012). When stressors do occur, those with higher levels of proactive coping are able to maintain their emotional functioning better than those with lower levels of proactive coping (Polk et al., 2020). Within the context of the COVID-19 pandemic, individuals who are at high risk of exposure to the virus, HCPs, could particularly benefit from engaging in proactive coping strategies in an effort to prevent exposure to future stressors. Indeed, we know from our past work that older adults, who are vulnerable to the effects of the virus, had lower levels of stress when they were high in proactive coping (Pearman et al., 2020).

This study is designed to examine the experiences of HCPs in the United States during this pandemic. Data collection took place between March 20 and May 14, 2020, a timeframe when the United States experienced a spike in new coronavirus cases, which limited the availability of important medical resources including appropriate personal protective equipment, and put tremendous strain on the nation’s HCPs. The sample is derived from a larger online study focused on individuals’ psychological and behavioral responses to COVID-19 (Pearman et al., 2020). In the current study, we specifically examine the following variables: stress related to COVID-19, anxiety about developing COVID-19, depressive symptoms, current general anxiety, past and future appraisals of stress related to COVID-19, perceived health and health-related concern, tiredness, control beliefs (mastery and constraint), and proactive coping in a sample of HCPs and age-matched controls. We hypothesized that HCPs would show significantly more challenges on our measures of stress, mental and physical health issues, control, and coping.

## Methods

### Participants

Amazon Mechanical Turk (mturk.com) was used to recruit participants for a larger study on the impact of COVID-19. MTurk is an international online crowdsourcing panel administered by Amazon and used here for collecting data. Potential participants responded to the description: *The purpose of this study is to examine how people living across the United States are reacting to the current COVID-19 pandemic. Select the link below to complete the 30-min survey*. Participant requirements for the current study were as follows: 18 years of age or older, living in the United States, native English-speakers and free from a dementia diagnosis. Once recruited and consented (see section “Procedure”), the participants completed the survey through the Qualtrics platform which is an online survey tool. The sample for the larger study consisted of 1,000 participants. Participants answered “Yes” or “No” to the question, “Are you a HCP?” Participants for the current study included all participants who answered “Yes” to this question as well as age-matched controls drawn from the same dataset. Because of concerns regarding age differences in our health indicators, we age-matched the controls. The final sample included 90 HCPs and 90 age-matched controls (*M*_age_ = 34.72 years, *SD* = 9.84, range = 23–67) from 35 states across the United States. Sample characteristics, including type of HCP, are reported in Table 1.

**TABLE 1:** Sample characteristics by group (in valid percentages).

Variables	Healthcare professionals (*n* = 90) (%)	Matched sample (*n* = 90) (%)
**Gender:**		
Men	54.4	54.4
Women	45.6	45.6
**Degree:**		
GED	0	1.1
High school graduate	0	4.4
Elementary/middle school	1.1	0
Two year college, vocational school, associate’s degree	1.1	6.7
Some college but no degree	6.7	12.2
Bachelor’s degree (e.g., BA, BS, BFA)	56.7	57.8
Some graduate school but no degree	1.1	1.1
Master’s degree (e.g., MA, MS, MPH)	27.8	12.2
Ph.D., EdD, MD, DDS, JD, other professional degree	5.6	4.4
**Race:**		
Asian	5.6	4.4
Black or African American	15.6	12.2
Native Hawaiian	2.2	0
White	75.6	81.1
More than one race	0	2.2
I do not wish to answer	1.1	0
**Healthcare occupation:**		
Nurse	13.3	
Physician	36.7	
Occupational therapy	2.2	
Physical therapy	4.4	
Technician	24.4	
Nursing assistant	4.4	
Other	11.1	
Not specified	3.3	

*Other Healthcare Occupations include *n* = 1 Administration, *n* = 1 Facility Manager, *n* = 1 Legal Operations, *n* = 1 Counselor, *n* = 1 Exercise Physiologist, *n* = 1 Health Insurance, *n* = 1 Medical Student, *n* = 2 Optometry, *n* = 1 Registered Dental Hygienist.*

### Procedure

Informed consent was obtained online; participants who wished to participate in the study indicated electronically that they read and understood the study procedures. After indicating interest, participants were provided a Qualtrics survey link on MTurk between March 20, 2020 and May 14, 2020, which was the time period that encompassed the majority of stay-at-home orders as well as many peaks in hospitalizations and death from COVID-19 in the United States. Human intelligence tasks (HITS) were released approximately every 3 days on MTurk to promote continued enrollment and survey completion throughout the 6 weeks of data collection. Participants were compensated $3.00 for completing the 30-min survey. The study was approved by the Georgia Institute of Technology Institutional Review Board.

### Measures

#### Demographics

Participants indicated their year of birth, gender, their education from a checklist (e.g., GED, Associates), and their race. HCPs were also asked to report the specific profession within the healthcare field from a checklist (see Table 1).

#### COVID-19 Anxiety

Participants indicated their level of anxiety related to contracting coronavirus by answering the question, “How anxious are you about developing (COVID-19)?” on a 1 (*not at all anxious*) to 5 (*very anxious*) scale.

#### COVID-19 Stress

On a 1 (*not at all*) to 5 (*extremely*) scale, participants indicated their level of stress by answering the question, “How stressed are you about the COVID-19 outbreak?”

#### Depressive Symptoms

Participants completed the 15-item Geriatric Depression Scale Short Form (GDS) (Yesavage, 1988). The GDS is a self-report screening tool that examines depressive symptoms. Reflecting over the past week, participants respond “Yes” or “No” to each item. An example item includes, “Do you feel that your situation is helpless?” The scale has been shown to have good diagnostic sensitivity and specificity for adults across the adult lifespan (Guerin et al., 2018). The scale was not used for diagnostic purposes in this study, but higher scores indicate greater depressive symptoms (α = 0.81).

#### Current Anxiety

Ten state anxiety items from the State-Trait Anxiety Inventory (Spielberger et al., 1983) were rated on a four-point scale ranging from 1 (*not at all*) to 4 (*very much so*). Participants indicated how they were feeling in the current moment. Example items include “I am tense” and “I feel frightened.” Five items were reverse coded. A mean was calculated across the 10 items with higher scores indicating more state anxiety (α = 0.88).

#### Health

Participants self-rated their health on a five-point scale ranging from 1 (*poor*) to 5 (*excellent*) by answering the question, “How would you rate your overall health?” In addition, participants rated their health concern on a 1 (*no concern*) to 5 (*very serious concern*) scale, responding to the question, “How much concern/distress do you feel about your health at this time?” Both items were included in analyses as one focuses on current health status while the other focuses more specifically on how concerned the individual is about their health.

#### Tiredness

On a five-point scale ranging from 1 (*not at all tired*) to 5 (*very tired*), participants were asked “In general, how tired are you right now?”

#### Control Beliefs

Control beliefs were measured using the mastery (four items, α = 0.84) and constraint (eight items, α = 0.95) scales from the Sense of Control Scales from the Midlife Development Inventory (Lachman and Weaver, 1998a). On a 1 (*strongly disagree*) to 7 (*strongly agree*) scale, participants rated their agreement with statements such as “What happens in my life is often beyond my control” (constraint) and “I can do just about anything I really set my mind to” (mastery).

#### Proactive Coping

The Proactive Coping Scale (Aspinwall et al., 2005) includes six items rated on a five-point scale ranging from 1 (*strongly disagree*) to 5 (*strongly agree*). An example item includes, “I prepare for adverse events.” One item was reverse coded. Higher scores indicate more proactive coping (α = 0.71).

#### Stress Appraisals

On a four-point scale ranging from 1 (*not at all*) to 4 (*a lot*), participants rated the extent to which COVID-19 affected different areas of their lives in the past 24 h (past appraisal, α = 0.84) as well as the extent to which they expected COVID-19 to affect their lives in the next 24 h (future appraisal, α = 0.87). Example items include, “Your physical health or safety?” and “Your plans for the future?” (Lazarus, 2006). Items were scored so that higher scores indicate COVID-19 having a greater impact on one’s life.

### Statistical Analysis

All data analyses were performed using SPSS version 26 (IBM Corp.). The significance level was set at α = 0.05 and all tests were two-tailed. A MANOVA was conducted with education (degree) as a covariate and HCP (0 = *no*, 1 = *yes*) as the independent variable and COVID-19 stress and anxiety, depressive symptoms, current anxiety, self-reported health, health concern, tiredness, mastery, constraint, proactive coping and appraisal (past and future) as continuous dependent variables. Because healthcare positions commonly require postsecondary education, education was included as a covariate to account for group differences. Significant multivariate tests were followed up with tests of between-subjects effects for each dependent variable individually.

## Results

There were no significant differences between HCPs and the control group on gender [χ^2^ (1, *N* = 180) = 0.00, *p* = 1.00] or race [χ^2^ (5, *N* = 180) = 5.65, *p* = 0.34]. As expected, there were significant differences on education [χ^2^ (8, *N* = 180) = 16.61, *p* = 0.03] such that HCPs had more education than non-HCPs. Results from the MANOVA (Table 2) controlling for education show that HCPs reported significantly higher levels of depressive symptoms, current anxiety, concern about their health, tiredness, constraint, and past and future appraisal of COVID-related stress, but lower levels of proactive coping compared to non-HCPs (Pillai’s Trace = 0.28, *F*(12,160) = 5.29, *p* < 0.001, η^2^ = 0.28). Of note, there were also no significant group differences on COVID-related stress or on the specific anxiety of developing COVID-19.

**TABLE 2:** MANOVA results with means and standard deviations by group.

	Healthcare Professional (*n* = 85)		Matched Sample (*n* = 89)			
Variables	Mean	*SD*	Mean	*SD*	*P*	η^2^
COVID-19 anxiety	3.25	1.46	3.20	1.48	0.74	0.001
COVID-19 stress	3.52	1.12	3.22	1.24	0.22	0.01
Depressive symptoms	6.49	3.23	4.58	3.75	0.001	0.06
Current anxiety	2.32	0.54	2.01	0.77	0.003	0.05
Self-rated health	3.92	0.93	3.75	1.00	0.38	0.004
Health concern	3.40	1.25	2.53	1.16	<0.001	0.11
Tiredness	2.93	1.29	1.85	0.96	<0.001	0.18
Mastery	5.11	1.01	5.28	1.17	0.27	0.01
Constraint	4.64	1.36	3.28	1.50	<0.001	0.16
Proactive coping	3.62	0.58	4.08	0.63	<0.001	0.12
Appraisal (past 24 h)	2.61	0.63	2.22	0.74	0.002	0.05
Appraisal (next 24 h)	2.60	0.66	2.20	0.81	0.002	0.05

*η^2^ = partial eta squared. Multivariate Test Results: Pillai’s Trace = 0.28, *F*(12,160) = 5.29, *p* < 0.001, η^2^ = 0.28, observed power = 1.00.*

## Discussion

This study is a timely look into the experiences of HCPs across the United States during the COVID-19 pandemic. Using an age-matched comparison group, the HCPs were significantly more depressed and generally anxious than the non-HCPs during the first months of the pandemic. In line with Shechter et al. (2020) who documented high rates of lack of control and sleep disturbances within HCPs in New York City, our results show that HCPs across the United States had significantly higher rates of lack of control and tiredness compared to controls. Additionally, the HCP group on average fell into the clinically depressed range on the GDS (Guerin et al., 2018). While some of the other findings (e.g., fatigue) may represent the nature of professional differences sometimes seen between HCPs and other professions in non-pandemic times (Dyrbye et al., 2014), meeting the criteria for depressive disorder should not. We believe that the heightened level of depressive symptoms in HCPs may be due to not just occupational differences but occupational differences during a pandemic. Clearly, this is of concern not just for understanding and, perhaps, helping the current situation but also to look ahead to the potential lasting influence of this experience (see Maunder et al., 2006; Lee et al., 2007). It is well-understood that the long-term consequences of depression and anxiety can create enduring negative impacts (Sareen et al., 2005; Musliner et al., 2016). Finding ways to intervene and support HCPs, such as cognitive behavioral therapy or support groups, will be an important goal to healthcare systems and workplaces now and in the future.

In addition to increased general anxiety and depressive symptoms, HCPs were more tired and more concerned about their health than the age-matched controls. There are many possible reasons for the health concerns of HCPs during this pandemic (Centers for Disease Control, 2020). To start, HCPs are more likely to be exposed to COVID-19 which increases HCP’s health risk. Other health risks include long work hours and mental and physical exhaustion (Shanafelt et al., 2020; The Lancet, 2020). It is not surprising therefore that the HCPs also have higher perceived constraints and are more tired. The real experiences in healthcare settings during the pandemic may present HCPs with what seem like insurmountable pressure when it comes to finding ways to accomplish their goals both in terms of maintaining their own health and well-being. Helping HCPs find ways to differentiate between immovable constraints, such as personal protective equipment deficits, and possible malleable constraints, such as feeling as though there is no opportunity to engage in self-care, may be a possible avenue for buoying the well-being of HCPs (De Raedt and Hooley, 2016).

Along these same lines, the HCPs showed lower proactive coping and fewer resources to dedicate to adaptive coping behaviors. We know from past work that proactive coping (Polk et al., 2020) and control beliefs (Neupert et al., 2007) are key ingredients for resilient stress responses, representing potential targets for intervention. For instance, Stauder et al. (2017, 2018) found that using coping skills training with employees from work-environments that were stressful, but unchanging, helped reduce stress and improve well-being.

Although statistically equivalent on COVID-19-related stress and anxiety, the HCPs in the current study scored significantly higher on both current and future stress appraisal when compared to controls. In their real-time study of work stress in nurses, Johnston et al. (2016) showed that appraisals of stress were more predictive of psychological and physiological reactivity than the actual tasks being performed. In addition, the perceived reward for the work actually helped reduce stress. Given the high levels of stress appraisal both current and future in our sample, it may be beneficial during this time of crisis to help HCPs recognize and focus on the reward of their work as a means of managing negative stress appraisals.

We acknowledge several limitations in this study. The observational design limits our ability to make causal conclusions. Future longitudinal studies should examine the long-term impact of this pandemic on the mental health of HCPs. We also do not know the extent to which the HCPs in the sample are serving on the frontlines of the pandemic. However, given that the HCPs showed significant differences on most of our measures of interest, it is likely that our effects actually underestimate the experiences of frontline workers. In addition, Smereka and Szarpak (2020) note that COVID-19 is an ongoing challenge for all HCPs, not just the frontline workers. Another potential limitation is that the professions of the control group nor the hours worked by either group were collected so we are unable to make finer distinctions between the experiences of HCP and the others. We do know, however, that the two groups are statistically equivalent in their stress and anxiety related to the pandemic, so we are reasonably confident that the differences that we do see in our study are associated with healthcare profession status. We encourage future work that seeks to further explore potential differences between professions, but note that our results suggest that all HCPs are at risk for decreased well-being, perceived control, and coping resources during the COVID-19 pandemic. Finally, our sample was not random or nationally representative and was restricted to those living in the United States, the current epicenter of the pandemic. HCPs’ experiences during the COVID-19 pandemic could differ for those living and working in countries outside of the United States.

In conclusion, our results suggest that COVID-19 may function as an occupational hazard for HCPs (Godderis et al., 2020) because we found evidence of higher levels of anxiety and depressive symptoms, more tiredness and concern for their health, and more severe stress appraisals of COVID-19, along with lower levels of perceived control and coping compared to age-matched controls. Across a wide array of indicators, HCPs appear to be at increased risk for mental health challenges. In addition, given that previous studies during other pandemics have shown lasting impacts of service during this time, including reduced workforce participation and increased traumatic symptomatology, this is a critical issue to address. We encourage efforts to intervene that can provide relief now and in the future.

## Data Availability Statement

The dataset presented in this article is not readily available because data sharing options were not included in consent documents. Requests to access the datasets should be directed to AP, ann.pearman@psych.gatech.edu.

## Ethics Statement

This study involved human participants was reviewed and approved by Georgia Institute of Technology Office Research Integrity Assurance – Institutional Review Board (Protocol # H20141). Written informed consent for participation was not required for this study in accordance with the national legislation and the institutional requirements for exempt studies.

## Author Contributions

AP designed the study and funded it out of her internal funds and a grant both from Georgia Tech, as well as manuscript writing. MH managed the online portion of the project and the data, wrote the methods, helped to prepare the references, and helped with primary prose. ES helped with data analyses, created the tables, helped to prepare the references, and helped with primary prose. SN helped with study design, primary data analyses, as well as manuscript writing. All authors contributed to the article and approved the submitted version.

## Conflict of Interest

The authors declare that the research was conducted in the absence of any commercial or financial relationships that could be construed as a potential conflict of interest.
